# Human Metapneumovirus Induces IRF1 *via* TANK-Binding Kinase 1 and Type I IFN

**DOI:** 10.3389/fimmu.2021.563336

**Published:** 2021-06-24

**Authors:** Simon Loevenich, Alix S. Spahn, Kristin Rian, Victor Boyartchuk, Marit Walbye Anthonsen

**Affiliations:** ^1^ Department of Clinical and Molecular Medicine (IKOM), Norwegian University of Science and Technology (NTNU), Trondheim, Norway; ^2^ Clinic of Surgery, St Olav Hospital HF, Trondheim, Norway; ^3^ Centre for Integrative Genetics, Department of Animal and Aquacultural Sciences, Faculty of Biosciences, Norwegian University of Life Sciences, Ås, Norway

**Keywords:** human macrophages, innate immune response, antiviral response, interferon, human metapneumovirus, interferon regulatory factor 1, nuclear factor-kB, TANK-binding kinase 1

## Abstract

The innate immune and host-protective responses to viruses, such as the airway pathogen human metapneumovirus (HMPV), depend on interferons (IFNs) that is induced through TANK-binding kinase 1 (TBK1) and IFN regulatory factors (IRFs). The transcription factor IRF1 is important for host resistance against several viruses and has a key role in induction of IFN-λ at mucosal surfaces. In most cell types IRF1 is expressed at very low levels, but its mRNA is rapidly induced when the demand for IRF1 activity arises. Despite general recognition of the importance of IRF1 to antiviral responses, the molecular mechanisms by which IRF1 is regulated during viral infections are not well understood. Here we identify the serine/threonine kinase TBK1 and IFN-β as critical regulators of IRF1 mRNA and protein levels in human monocyte-derived macrophages. We find that inhibition of TBK1 activity either by the semi-selective TBK1/IKKε inhibitor BX795 or by siRNA-mediated knockdown abrogates HMPV-induced expression of IRF1. Moreover, we show that canonical NF-κB signaling is involved in IRF1 induction and that the TBK1/IKKε inhibitor BX795, but not siTBK1 treatment, impairs HMPV-induced phosphorylation of the NF-κB subunit p65. At later time-points of the infection, IRF1 expression depended heavily on IFN-β-mediated signaling *via* the IFNAR-STAT1 pathway. Hence, our results suggest that TBK1 activation and TBK1/IKKε-mediated phosphorylation of the NF-κB subunit p65 control transcription of IRF1. Our study identifies a novel mechanism for IRF1 induction in response to viral infection of human macrophages that could be relevant not only to defense against HMPV, but also to other viral, bacterial and fungal pathogens.

## Introduction

Members of the interferon (IFN) regulatory factors (IRFs), a family of transcription factors, are activated downstream of pattern recognition receptors (PRRs) and are crucial for immunoregulation and stimulation of innate and adaptive immune responses (1). IRF1 was the first IRF identified to drive transcription of type I IFN genes and to be essential for host defense against a wide range of viruses, including influenza virus, human rhinovirus (HRV), varicella-zoster virus (VZV), respiratory syncytial virus (RSV), flaviviruses and West Nile virus (1–5). Subsequently, in addition to its role in control of antiviral defense, IRF1 has been found to have diverse functions, i.e. in regulation of apoptosis, DNA damage responses, tumor suppression and in shaping adaptive CD8(+) T cell immune responses (5, 6). The viral defense mechanisms triggered by IRF1 rely on induction of a large panel of genes that are important for mounting effective innate and adaptive immunity, including type III IFNs (IFN-λs) (7, 8). Type III IFNs are critical for antiviral defense at epithelial barrier sites that are important for airway viruses (9–11). In resting cells, IRF1 is expressed at very low levels in most cell types and the protein is highly unstable with a half-life of around 30 minutes (12, 13). Hence, stimulus-induced transcription of IRF1 is required to increase cellular levels of IRF1 to enable IRF1-dependent gene expression. Despite the critical importance of IRF1 for antiviral defense mechanisms the molecular mechanisms by which induction of IRF1 is regulated by viruses are largely unknown. IRF1 is highly upregulated upon viral infections, e.g. in response to influenza virus, RSV and VZV (3, 4, 14) as well as by IFN treatment (15, 16). Upon treatment with IFN-γ- or IFN-α/β the molecular regulation at the IRF1 promoter has been well defined and entails the DNA elements GAS (γ-activated sequence), which binds STAT1-STAT1 homo- or STAT1-STAT2 heterodimers, and nuclear factor-κB (NF-κB) binding sites (17–20). In addition, upon infection with the airway viruses HRV and RSV and after treatment with TNF, NF-κB activation (and the NF-κB element in the IRF1 promoter) was suggested to control induction of IRF1 (21–23).

TANK-binding kinase 1 (TBK1) is a non-canonical IκB kinase that in addition to its critical role in induction of type I IFNs regulates inflammation-related processes such as neuroinflammation and autophagy (24–26). In the process of type I IFN induction, TBK1 has been found to directly phosphorylate and activate the two IRF family-members IRF3 and IRF7, thereby inducing IRF dimerization and nuclear translocation to stimulate gene transcription (24, 25). While the activation mechanism of IRF3 and IRF7 by TBK1 has been intensively studied, the mechanism by which TBK1 affects IRF1 function has to the best of our knowledge not been reported.

Human metapneumovirus (HMPV, genus Metapneumovirus), belonging to the newly formed *Pneumoviridae* family, is related to RSV, is a significant cause of airway infections and may cause serious illness in infants and immunocompromised individuals (27). Several PRRs have been reported to be involved in innate immune sensing of HMPV, e.g. the RIG-I-like receptors (RLR) RIG-I and MDA5 and the Toll-like receptors TLR3 and TLR7 (27). The relative contribution of these PRRs depends on the cell type infected (27, 28). Also, TLR4 have been reported to contribute to cytokine induction by HMPV (29). While one study found that HMPV enhances IRF1 levels and IRF1 nuclear accumulation in airway epithelial cells (30), the molecular mechanisms underlying HMPV-stimulated IRF1 transcription have not been identified. Since IRF1 is required for induction of type III IFNs, that are critical for antiviral defense against several viruses including HMPV (11, 31), we chose to characterize HMPV-mediated induction of IRF1 expression. Macrophages are important immune cells that contribute to airway virus defense mechanisms and pathogenesis related to influenza virus, RSV and HMPV (32–34) and HMPV-mediated immune mechanisms in human macrophages are largely unexplored. In this study, we aimed to determine if the innate immune kinase TBK1 controls IRF1 transcription in human monocyte-derived macrophages (MDMs). Our results show that that TBK1 mediates HMPV-stimulated induction of IRF1, in a mechanism involving NF-κB activation and type I IFN-mediated STAT1 activation that lead to induction of IRF1.

## Methods

### Virus Propagation

The clinical HMPV isolate NL/17/00 (A2) was kindly provided by ViroNovative and B. van den Hoogen (Erasmus MC, Rotterdam). LLC-MK2 cells were inoculated with HMPV at a multiplicity of infection (MOI) of 0.01 in OptiMEM containing 2% FBS, 20 µg/mL gentamicin and 0.7 nM glutamine. After 7-8 days, the virus was harvested by freeze-thawing at -80 ⁰C, followed by purification on a 20% sucrose cushion and resuspension in OptiMEM (2% FBS). The virus titer was determined using a cell-based immunoassay. To that end, purified virus particles were serially diluted (log10) on monolayers of LLC-MK2 cells in 96-well flat-bottom plates. After four days, cells were washed, stained with LIGHT DIAGNOSTICS™ HMPV direct fluorescence assay (Merck Millipore) and foci forming units determined by manual counting. For inactivation, virus was irradiated with UV light for 1 h at 4°C.

### Cell Culture

Monocytes were obtained from fresh buffy coats of healthy donors (blood bank of St. Olavs Hospital, Trondheim). In short, mononuclear cells were isolated using gradient centrifugation with Lymphoprep™ (Axis-Shield). Isolated cells were seeded in RPMI 1640 medium supplemented with 10 % human serum, 0.34 mM L-glutamine, 10 µg/mL gentamicin. After 90 min non-adherent cells were washed away. Monocytes were differentiated to macrophages for 14 days in RPMI 1640 supplemented with 10 % human serum, 0.34 mM L-glutamine, 10 µg/mL gentamicin and 10 ng/mL macrophage colony-stimulating factor.

### *In Vitro* HMPV Infection

If not indicated otherwise, cells were infected with HMPV A2 at MOI 1 in OptiMEM containing 2% FBS, 20 µg/mL gentamicin and 0.7 nM glutamine. Cells were incubated with the virus for the indicated times.

### Recombinant Interferons, Neutralizing Antibodies, and Inhibitors

Human recIFN-β and -λ1 were purchased from Peprotech. NIFNλR1 (MMHLR-1) and nIFNAR2 (MMHAR-2; interferon-α/β receptor 2) were purchased from PBL. Cells were incubated with nIFNλR1 (10 µg/mL) or nIFNAR (10 µg/mL) 30 min before infection with virus. RecIFN-β (1000 U/mL) or recIFN- λ1 (1 µg/mL) were added 15 h after infection with virus for a total of 3 h. Cells were incubated with the pharmacological TBK1 inhibitor BX795 (10 µM; InvivoGen) 30 min prior to infection with HMPV if not indicated otherwise. Cells were incubated with either 5 µM or 10 µM S-Ruxolitinib 2 h prior to infection with virus.

### RNA Interference

siRNAs were purchased from Qiagen (AllStar Negative Control) and Ambion (IRF1, RelA and TBK1), respectively. siRNA duplexes were transfected into MDMs (day 9 after M-CSF addition) using Lipofectamine RNAiMAX siRNA transfection reagent (Thermo Fisher Scientific) and in according to the manufacturer’s instructions, yielding a final concentration of 10 or 20 nM siRNA. The medium was replaced after 24 hours and the transfection was repeated 3 days after the initial transfection. mTransfected cells were allowed to grow for 48 hours prior to HMPV infection or mock treatment and harvesting of cells for qRT-PCR or immunoblotting analysis.

### QRT-PCR Analysis

RNA isolation, cDNA synthesis and qRT-PCR analysis were performed as previously described (35). Gene expression was calculated relative to uninfected cells according to Livak et al. (36). The following primers were used: *viperin (fwd) TGCTTTTGCTTAAGGAAGCTG* and *viperin (rev) CAGGTATTCTCCCCGGTCTT; IKKϵ (fwd) CAAGCTGACAGACTTCGGCG* and *IKKϵ (rev) GTGATCCGCTACATGATCTC*. All other primer sequences have been published previously (35, 37). For analysis of HMPV vRNA expression, the following primers were used: *HMPV N-gene (fwd)* CATATAAGCATGCTATATTAAAAGAGTCTC, *HMPV N-gene (rev)* CCTATTTCTGCAGCATATTTGTAATCAG. Fold-change in HMPV vRNA expression was calculated relative to the indicated virus sample.

### Immunoblotting

Preparation of whole-cell lysates was performed as described previously (38). SDS-PAGE and immunoblotting of whole cell lysates were performed as previously described (38). Band intensities were normalized against a housekeeping protein (β-actin/GAPDH) using Image Studio (Licor). If not indicated otherwise, protein levels are presented as fold change relative to uninfected cells (“N.I.”). Nuclear and cytosolic fractionation of samples was performed based on a previously published protocol with modifications (39). Cells were detached by trypsination followed by scraping. Cells were washed in 30 volumes of PBS and centrifuged (5 min, 450 x g). The cell pellet was resupended in one packed cell volume (PCV) of ice-cold Buffer A + DTT with minimal pipetting and allowed to swell on ice for 15 min. The cells were lysed by slowly drawing the cell suspension into a 1 ml syringe with a 25-g 5/8 gauge needle and then rapidly expelling in a single stroke. This was repeated 5 times. The homogenate was centrifuged for 5 min at 12,000 x g at room temperature, yielding a crude nuclear pellet and a post nuclear supernatant. The post nuclear supernatant was processed to make a cytoplasmic extract by adding 0.11 PCV of Buffer B and spinning 5 min at 12,000 x g and 4°C. The crude nuclear pellet was resuspended in 0.67 PCV of ice-cold Buffer C containing 420 mM NaCl, then incubate on ice for 30 min. Nuclear debris was removed by centrifugation (5 min, 12.000 x g, 4°C). The supernatant containing the nuclear extract was snap-frozen in liquid nitrogen and stored at -80°C. SDS-PAGE and immunoblotting of nuclear fractions were performed as for whole cell lysates. Band intensities for nuclear fractions were normalized against medium and loading control (Histone 3). Changes in nuclear protein level are presented relative to the respective cytosolic levels. The following primary antibodies were purchased from Cell Signaling Technology if not indicated otherwise: β-actin (Sigma Aldrich), GAPDH, histone 3 (Abcam), HMPV Nucleoprotein (Abcam), IRF1, IRF3, p-IRF3(S396), p65, p-p65(Ser536), p-STAT1(Tyr701), STAT1, p-TBK1(Ser172), TBK1, α-tubulin (Santa Cruz Biotechnology).

### Statistical Analysis

Data are representative of 3-5 independent biological experiments, unless otherwise stated. A two-tailed Student’s t-test was used for statistical analysis of single comparisons. Differences were considered significant when p ≤ 0.05 (∗), very significant when p ≤ 0.01 (∗∗), highly significant when p ≤ 0.001 (∗∗∗), and extremely significant when p ≤ 0.001 (∗∗∗∗). Comparisons of one or more variables between multiple groups were analyzed by two-way ANOVA followed by *post hoc* Tukey’s honest significance test. Differences were considered significant when p ≤ 0.05 (∗), very significant when p ≤ 0.01 (∗∗), highly significant when p ≤ 0.001 (∗∗∗), and extremely significant when p ≤ 0.001 (∗∗∗∗).

## Results

### IRF1 Is Induced by HMPV Infection and Is Critical for IFN-λ and IFN-β Transcription

**Figure 1:**
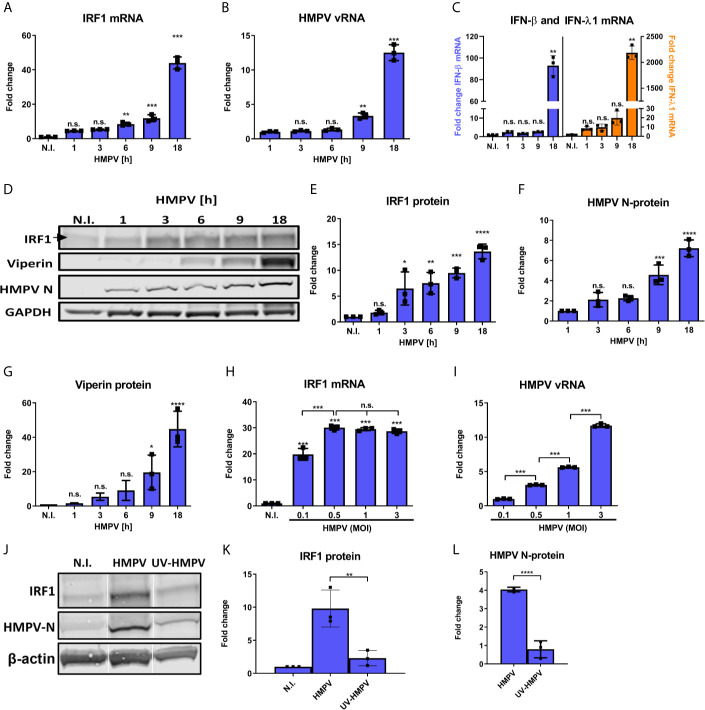
HMPV induces IRF1 expression from early timepoints of infection. **(A–G)** Human MDMs were infected for the indicated timepoints with HMPV or treated with medium (N.I.). Expression of IRF1 mRNA **(A)**, HMPV vRNA **(B)**, IFN-β and IFN-λ1 mRNA **(C)** was analyzed by qRT-PCR. Error bars represent SD of three technical replicates. **(D–G)** Whole cell lysates were prepared and protein levels of IRF1 **(E)**, HMPV N-protein **(F)** and viperin **(G)** were analyzed by immunoblotting. Protein levels were quantified by normalization of band intensities against GAPDH and are presented as fold change relative to uninfected cells (“N.I.”). **(H, I)** Human MDMs were infected for 18 h with HMPV at the indicated MOI or treated with medium (“N.I.”). Expression of IRF1 mRNA **(H)** and HMPV vRNA **(I)** was analyzed by qRT-PCR. Error bars represent SD of three technical replicates. **(J–L)** Human MDMs were infected for 18 h with HMPV or UV-inactivated HMPV or treated with medium (“N.I.”). Expression of IRF1 **(K)** and HMPV N-protein **(L)** were determined by immunoblotting. Statistical analysis: One-way ANOVA followed by Tukey’s multiple comparison test: ^∗^p < 0.05; ^∗∗^p < 0.01; ^∗∗∗^p < 0.001; ^∗∗∗∗^p < 0.0001; n.s., not significant.

To characterize IRF1 levels in human monocyte-derived macrophages (MDMs) in response to HMPV we analyzed IRF1 expression kinetics. To achieve this, IRF1 mRNA and protein levels were measured in MDMs infected with HMPV at different timepoints ranging from 1 to 18 h. Consistent with previous reports (3, 4, 14), IRF1 levels in uninfected MDMs were low and both IRF1 mRNA and protein expression were induced by HMPV infection. Induction of IRF1 mRNA and protein were detectable as early as 1-3 hours post infection (h.p.i.) and increased markedly up to 18 h.p.i. (**Figures 1A, E**). IRF1 expression correlated well with HMPV replication, as monitored by increased viral RNA (vRNA) and HMPV nucleoprotein (N-protein) (**Figures 1B, D, F**). Moreover, we compared IRF1 induction by HMPV to that of LPS and polyIC and found that LPS induces IRF1 more potently than HMPV, while polyIC induces IRF1 to the similar extents as HMPV (**Figure S1**).

Both IFN-β and IFN-λ1 mRNA was induced by HMPV in a time-dependent manner following increase in HMPV vRNA, with higher fold-induction of IFN-λ1 than of IFN-β (**Figure 1C**). To determine the specificity of the observed IRF1 induction, we also examined the time-dependent expression of viperin (virus inhibitory protein, endoplasmic reticulum-associated, IFN-inducible), another IFN-stimulated gene (ISG). Viperin is induced by type I, II, and III IFNs and after infection with a broad range of DNA and RNA viruses (40). Compared to IRF1, viperin was markedly induced at later timepoints of the HMPV infection (**Figure 1G**).

To determine the effect of viral dose, we compared IRF1 mRNA expression in cells infected at different MOIs. We found that vRNA levels increased proportionally with MOI, while IRF1 mRNA induction was maximal over the range from 0.5-3 MOI (**Figures 1H, I**). The reason for the relative higher IRF1 expression at lower vRNA levels could be related to earlier reports demonstrating higher JAK/STAT signaling through the IFNAR in cell cultures infected with lower titers of Sendai virus (a paramyxovirus) (41). We determined that induction of ISG54 (IFIT2) followed the same pattern as IRF1 at different MOIs (**Figure S2**). UV-inactivation of HMPV led to markedly reduced induction of IRF1 (**Figures 1J, K**) suggesting that viral replication is required for efficient stimulation of IRF1 expression. We also confirmed that at 18 h treatment levels of HMPV N-protein of UV-inactivated HMPV was low compared to HMPV (**Figures 1J, L**).

**Figure 2:**
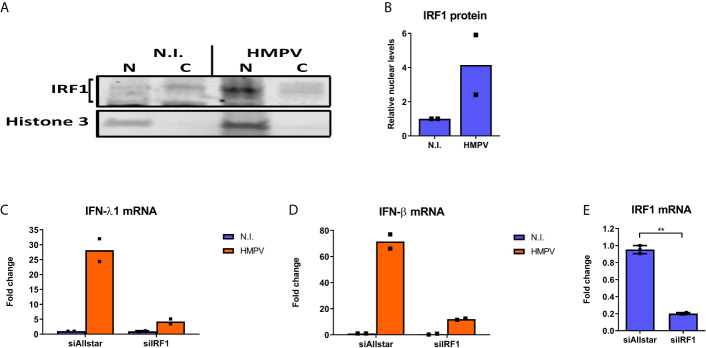
HMPV stimulates IRF1 nuclear translocation and IRF1-dependent IFN-λ induction. **(A, B)** Human MDMs were infected with HMPV for 18 h. Nuclear and cytosolic cell lysates were prepared and protein levels of IRF1 and histone 3 analyzed by immunoblotting. Changes in nuclear IRF1 protein level are presented relative to the respective cytosolic levels and to histone 3 levels **(B)**. **(C-E)** Human MDMs were transfected with IRF1 siRNA or control siRNA («Allstar») for 48h prior to infection with HMPV for 24 h and analysis of mRNA expression of IFN-λ1 **(C)**, IFN-β **(D)** and IRF1 **(E)** by qRT-PCR. Statistical analysis: Two-way ANOVA with Bonferroni correction Two-tailed Student’s t-test: ^∗∗^p < 0.01. Data are representative for two independent experiments.

Next we proceeded to probe if HMPV infection stimulates IRF1 activation and IRF1-dependent gene induction. To this end, we measured IRF1 nuclear translocation by immunoblotting of cytoplasmic and nuclear fractions of human MDMs that were infected with HMPV. HMPV infection led to increased IRF1 levels in nuclear fractions compared to cytoplasmic fractions (**Figures 2A, B**). Considering that IRF1 has recently been reported to be critical for IFN-λ1 induction by Dengue and Sendai virus (8), we used siRNA-mediated knockdown to determine if IRF1 levels affect IFN-λ1 and IFN-β mRNA induction by HMPV in MDMs. Knockdown of IRF1 reduced IRF1 mRNA levels to 20% (**Figure 2E**). Based on morphology of the cells (**Figure S3**) and LDH assay siRNA-transfection did not affect cell viability. Treatment with siIRF1 markedly reduced both IFN-λ1 and IFN-β mRNA (**Figures 2C, D**) suggesting the requirement for IRF1 in induction of both these IFNs at later timepoints of HMPV infection, similarly to what was found for Sendai virus infection (8). Collectively, these results indicate that in human MDMs, HMPV induces IRF1 expression resulting in increased IRF1 nuclear translocation and IFN-λ1 and IFN-β expression in an IRF1 dependent manner.

### TBK1 Activity Is Required for Induction of IRF1 by HMPV

**Figure 3:**
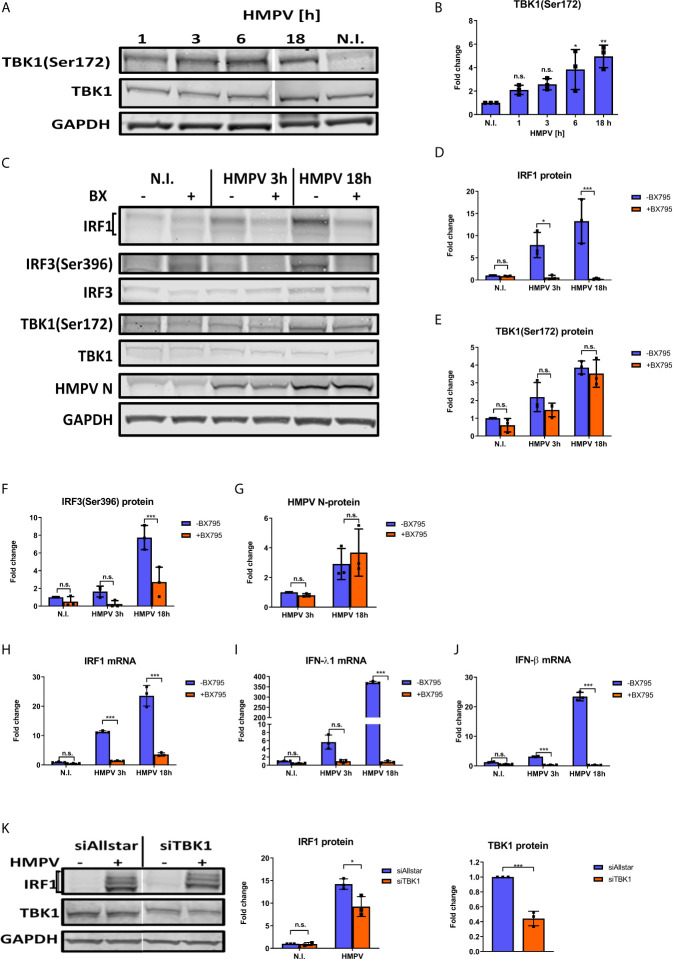
TBK1 is required for IRF1 expression in response to HMPV. **(A, B)** Human MDMs were infected for the indicated timepoints with HMPV or treated with medium (“N.I.”). Whole cell lysates were prepared and protein levels of TBK1 and p-TBK1(Ser172) were analyzed by immunoblotting. Protein levels were quantified by normalization of band intensities against GAPDH and are presented as fold change relative to uninfected cells (“N.I.”) **(B)**. **(C–J)** Human MDMs were pretreated with or without BX795 before infection with HMPV for the indicated timepoints. **(C–G)** Whole cell lysates were prepared and protein levels of IRF1, p-TBK1(Ser172), TBK1, p-IRF3(Ser396) and IRF3 were analyzed by immunoblotting. Protein levels were quantified by normalization of band intensities against GAPDH and are presented as fold change relative to uninfected cells (“N.I.”). **(H–J)** MRNA expression of IRF1, IFN-λ1 and IFN-β was determined by qRT-PCR. **(K)** MDMs were transfected with TBK1 siRNA or control siRNA («Allstar») prior to infection with HMPV for 18 hours. Whole cell lysates were prepared and protein levels of IRF1 and TBK1 were analyzed by immunoblotting. Protein levels were quantified by normalization against siAllstar N.I. cells and GAPDH levels. Statistical analysis: Two-way ANOVA with Tukey’s honest significance test: ^∗^p < 0.05; ^∗∗^p < 0.01; ^∗∗∗^p < 0.001; n.s., not significant.

TBK1 has been found to directly phosphorylate and activate the two IRF family-members IRF3 and IRF7, but the contribution of TBK1 to IRF1 activation and function has not been reported. To test our hypothesis that TBK1 mediates IRF1 induction in HMPV-infected cells, we initially measured if HMPV affected TBK1 Ser172-phosphorylation levels (required for TBK1 activation (24)) in human MDMs (42).. Interestingly, HMPV induced TBK1 Ser172-phosphorylation starting from early timepoints of (3-6 h.p.i.; showing statistical significance from 6 h.p.i) and continued to increase up to 18 h.p.i. (**Figures 3A, B**). To explore if TBK1 was necessary for HMPV-stimulated induction of IRF1 we first tested the effect of BX795, a potent and relatively specific inhibitor of TBK1 and its homolog IKKϵ (IκB kinase ϵ (42);). Human MDMs were preincubated BX795 prior to infection with HMPV for 3 or 18 h. Treatment with BX795 led to a marked decrease of HMPV-induced IRF1 protein (**Figures 3C, D**) and IRF1 mRNA levels (**Figure 3H**) both at 3 and 18 h.p.i. In contrast, the levels of HMPV were not markedly affected by BX795 treatment at these timepoints (**Figures 3C, G**). As reported by others (42, 43), we observed that BX795 failed to reduce TBK1S172 phosphorylation (**Figures 3C, E**), an observation suggested to be due to a negative feedback loop triggered by TBK1 (42). However, we note that in agreement with previous reports, treatment of MDMs with BX795 resulted in decreased phosphorylation of IRF3Ser396, a canonical TBK1 target (24) (**Figures 3C, F**). In line with this, treatment with BX795 abolished IFN-λ1 and IFN-β mRNA expression by HMPV (**Figures 3I, J**) showing that BX795 blocks the IRF3-IFN-β/λ axis. BX795 also reduces the activities of the kinases IKKε and PDK1 (42). Therefore, to specifically address the contribution of TBK1 to IRF1 induction we performed siRNA-mediated knockdown of TBK1 in MDMs prior to infection with HMPV. Knockdown of TBK1 reduced TBK1 protein levels to approximately 50% and this led to reduction of HMPV-stimulated IRF1 protein with about 40% (**Figure 3K**). Hence, BX795 reduced IRF1 levels to higher extent than siTBK1-treatment did. Taken together, these results demonstrate that TBK1 is activated by HMPV and contributes to HMPV-stimulated IRF1 expression in human MDMs.

### HMPV-Mediated IRF1 Expression Is Dependent on NF-κB Activation

**Figure 4:**
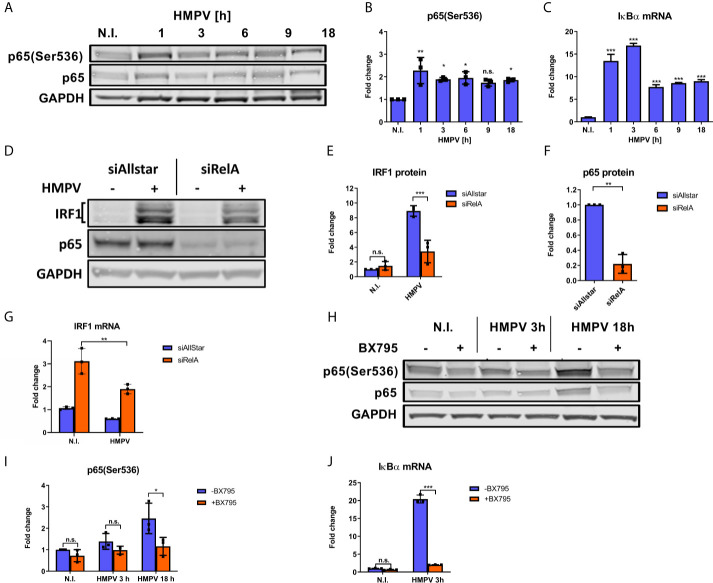
NF-κB is necessary for HMPV-induced IRF1 expression. **(A–C)** Human MDMs were infected for the indicated timepoints with HMPV or treated with medium (N.I.). **(A)** Whole cell lysates were prepared and protein levels of p65 and p-p65(Ser536) were analyzed by immunoblotting. **(B)** Protein levels were quantified by normalization of band intensities against GAPDH and are presented as fold change relative to uninfected cells (“N.I.”). **(C)** IκBα mRNA expression was determined by qRT-PCR. Error bars represent SD of three technical replicates. **(D–G)** MDMs were transfected with RelA siRNA or control siRNA («Allstar») prior to infection with HMPV. In **(D–F)** whole cell lysates were prepared and protein levels of IRF1, p65 and GAPDH were analyzed by immunoblotting. For **(E, F)**, protein levels were quantified by normalization of band intensities against siAllstar medium and the respective values for GAPDH. **(G)** IRF1 mRNA in siRNA-treated MDMs was determined by qRT-PCR. **(H–J)** Human MDMs were pretreated with or without BX795 before infection with HMPV for the indicated timepoints. Whole cell lysates were prepared and protein levels of p65, p-p65(Ser536) and IκBα were analyzed by immunoblotting **(H)**. Protein levels were quantified by normalization of band intensities against GAPDH and are presented as fold change relative to uninfected cells (“N.I.”; **I**). **(J)** IκBα mRNA expression was determined by qRT-PCR. Error bars represent SD of three technical replicates. Statistical analysis: Two-way ANOVA with Tukey’s honest significance test ^∗^p < 0.05; ^∗∗^p < 0.01; ^∗∗∗^p < 0.001; n.s., not significant.

IRF1 expression can be induced by the transcription factor NF-κB (12) and phosphorylation of the NF-κB p65 subunit at Ser536 is critical for its transcriptional activity (44). As a first step towards addressing NF-κB involvement in HMPV-induced IRF1 expression, we examined how HMPV infection affected p65 Ser536-phosphorylation. We found that HMPV induced phosphorylation of p65-Ser536 at all timepoints examined, with induction already at early timepoints of infection (**Figures 4A, B**). Activation of NF-κB is known to induce expression of the inhibitor of NF-κB α (IκB) (45). Hence, as an indirect measure of NF-κB activation we tested if HMPV triggered IκBα mRNA synthesis. At all timepoints examined (from 1 to 18 h), HMPV stimulated IκBα mRNA with the most prominent induction between 1 and 3 h.p.i. (**Figure 4C**). Thus, based on our observations of changes in p65 phosphorylation and IκBα mRNA expression, we conclude that HMPV activates the NF-κB pathway in human MDMs, already at early timepoints of HMPV infection.

**Figure 5:**
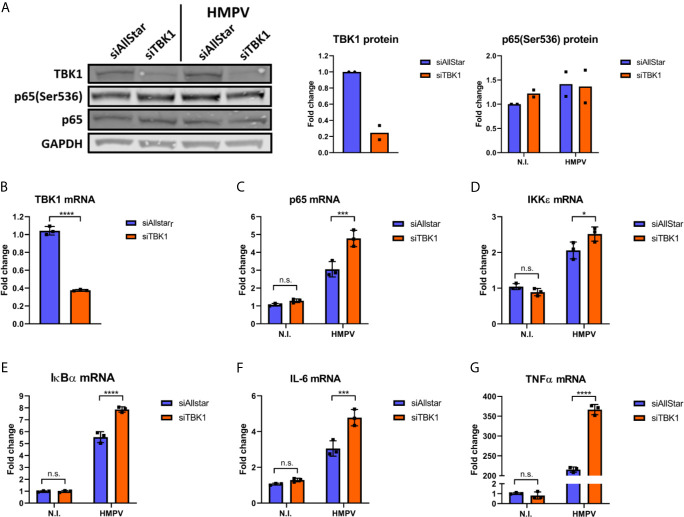
TBK1 is dispensable for HMPV-mediated p65 Ser536 phosphorylation and NF-κB activation. Human MDMs were transfected with TBK1 siRNA or control siRNA («Allstar») prior to infection with HMPV. **(A)** Whole cell lysates were prepared and protein levels of TBK1 and p-p65(Ser536) were analyzed by immunoblotting. **(B-G)** MRNA expression of TBK1 **(B)**, p65 **(C)**, IKKε **(D)**, IκBα **(E)**, IL-6 **(F)** and TNF **(G)** was determined by qRT-PCR. Statistical analysis: Two-way ANOVA with Tukey’s honest significance test: ^∗^p < 0.05; ^∗∗∗^p < 0.001; ^∗∗∗∗^p < 0.0001; n.s., not significant.

Our next goal was to determine whether NF-κB activation contributes to HMPV-stimulated IRF1 expression. To this end, we examined the effect of siRNA-mediated knockdown of RelA (which encodes p65) on HMPV-stimulated IRF1 expression. Knockdown of RelA markedly reduced p65 protein levels (**Figures 4D, F**) and attenuated the HMPV-induced increase of IRF1 protein levels to approximately 50% (**Figures 4D, E**). Corroborating this result, we also found that siRelA-treatment reduced IRF1 mRNA induction by HMPV (**Figure 4G**). Because TBK1 was originally identified as a mediator of p65 phosphorylation (46), we next asked if TBK1 affected p65 phosphorylation induced by HMPV. We found that treatment with BX795 strongly impaired HMPV-induced p65 phosphorylation at both 3 and 18 h.p.i. (**Figures 4H, I**). To test if TBK1 inhibition by BX795 also affected NF-κB transcriptional activity, we determined the effect of BX795 on HMPV-mediated IκBα mRNA expression and found that BX795 almost completely blocked HMPV-mediated IκBα mRNA expression at 3 h.p.i. (**Figure 4J**). As BX795 in addition to inhibiting TBK1 inhibits activation of its homolog IKKϵ, we examined the effect of siTBK1 on p65 Ser536-phosphorylation. We found that siTBK1-mediated repression of TBK1 failed to reduce p65 phosphorylation. However, we observed that p65 mRNA levels were increased (**Figures 5A–C**). Interestingly, it has been reported that upregulation of IKKϵ may be a mechanism to compensate for the loss of TBK1 activity in NF-κB activation (43, 47). To address this, we tested the effect of siTBK1 treatment on IKKε levels. Our results show that IKKε mRNA is induced by HMPV infection and is further increased when TBK1 is inhibited (**Figure 5D**). Moreover, we found that mRNAs of the NF-κB-regulated genes IkBα, IL-6 and TNF were increased in siTBK1-treated cells (**Figures 5E–G**), showing that IKKϵ upregulation coincided with NF-kB pathway activation. These results are consistent with recent findings by Balka et al. (43) showing that TBK1 and IKKϵ act redundantly in p65 phosphorylation and NF-κB activation. Since we found that NF-κB contributes to IRF1 expression by HMPV and as the NF-κB subunit RelA/p65 is also required for early IFN-β expression (48), we next evaluated the effect of siRelA on HMPV-stimulated IFN-β induction. siRelA-treatment reduced HMPV-stimulated IFN-β at 18 hours of infection (**Figure S4**). While little IFN-β was induced at 3 hours, IRF1 mRNA was stimulated to around 4-fold and this induction was inhibited by siRelA-treatment (**Figure S4**). Thus, our results indicate that HMPV-mediated IRF1 expression is dependent on NF-κB activation, that TBK1 contributes to NF-κB phosphorylation and transcriptional activation, and that NF-κB mediates IFN-β induction.

### HMPV-Induced Expression of IRF1 Is Dependent on Type I IFN Signaling

**Figure 6:**
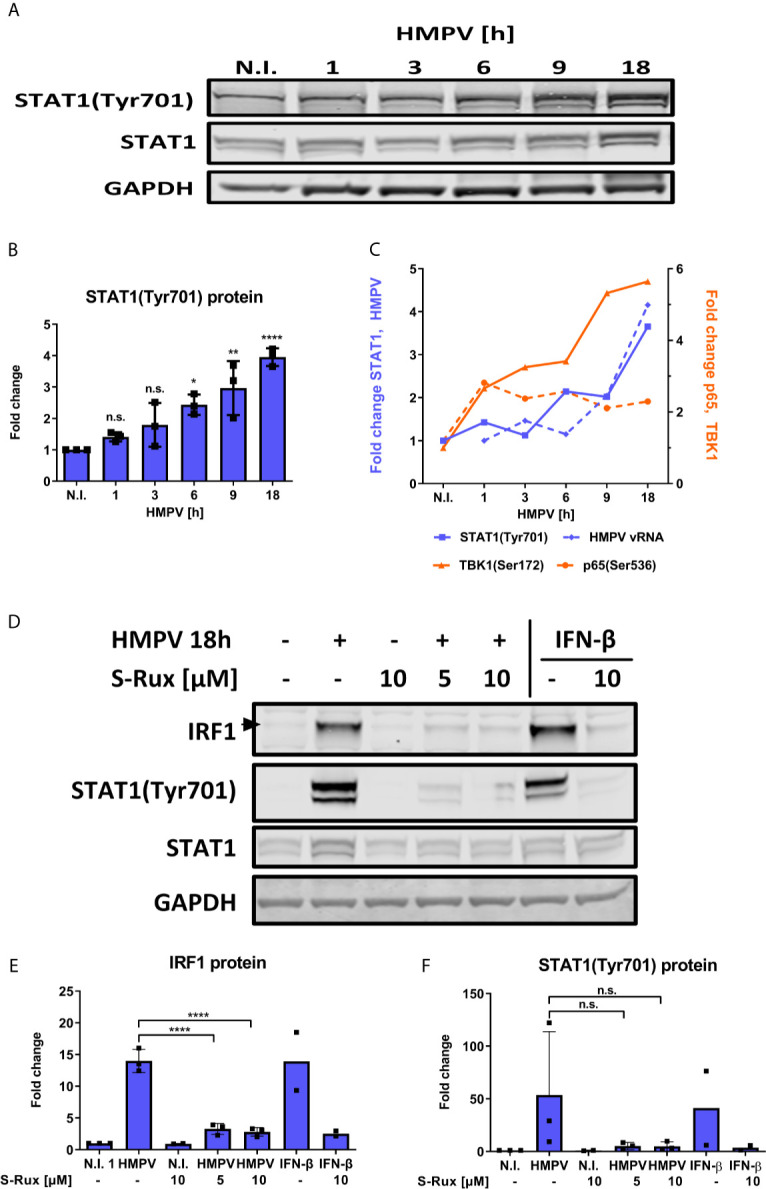
HMPV-induced expression of IRF1 is dependent on STAT1 activation. **(A, B)** Human MDMs were infected for the indicated timepoints with HMPV or treated with medium (“N.I.”). Whole cell lysates were prepared and protein levels of STAT1 and p-STAT1(Tyr701) were analyzed by immunoblotting. **(B)** Protein levels of p-STAT1(Tyr701) were quantified by normalization of band intensities against GAPDH and are presented as fold change relative to uninfected cells (“N.I.”). **(C)** Comparison of quantified p-STAT1(Tyr701), HMPV N-protein, p-TBK1(Ser172) and p-p65(Ser536) levels (presented in **Figures 1C**, **3A**, **4A** and **5A**). **(D)** Human MDMs were preincubated with the indicated concentration of S-Ruxolitinib prior to infection with HMPV (18 h) or treatment with IFN-β (3 h). Whole cell lysates were prepared and protein levels of IRF1, STAT1 and p-STAT1(Tyr701) were analyzed by immunoblotting. Protein levels of IRF1 **(E)** and p-STAT1(Tyr701) **(F)** were quantified by normalization of band intensities against GAPDH and are presented as fold change relative to uninfected cells (“N.I.”). Statistical analysis: Two-way ANOVA with Tukey’s honest significance test: ^∗^p < 0.05; ^∗∗^p < 0.01; ^∗∗∗∗^p < 0.0001. n.s., not significant.

In addition to NF-κB, IRF1 expression can be induced by STAT1 that is activated downstream of IFN-receptors (12). It was recently reported that robust STAT1 Tyr701 phosphorylation was required and sufficient for STAT1 binding to the IRF1 promoter in response to IFN-β (49). Because phosphorylation at Tyr701 is required for STAT1 activation and binding to the IRF1 promoter we measured levels of phosphoTyr701 STAT1 in HMPV-infected MDMs. From about 6 h.p.i., HMPV infection markedly increased STAT1 Tyr701-phosphorylation that continued to increase until 18 h.p.i. (**Figures 6A, B**). To visualize differences in kinetics of p65 and STAT1 phosphorylation we plotted the magnitude of change in phosphoprotein levels (by immunoblotting; **Figures 6A**, **4A**) in **Figure 6C**. By doing this, we illustrate that p65 phosphorylation occurs prior to STAT1 phosphorylation. Furthermore, to assess if inhibition of the STAT1 pathway affects IRF1 induction by HMPV, we measured the effect of the Janus family kinases JAK1 and JAK2 inhibitor ruxolitinib on IRF1 induction. JAK1/JAK2 are critical for STAT1-mediated gene induction and ruxolitinib is a potent inhibitor of STAT1-mediated gene expression (50–52). We found that in MDMs pretreated with either 5 or 10 µM ruxolitinib prior to HMPV infection induction of IRF1 protein was abrogated (**Figures 6D, E**). In addition, we confirmed that ruxolitinib blocked HMPV-stimulated phosphorylation of STAT1 at Tyr701 (**Figures 6D, F**). Ruxolitinib also blocked IRF1 expression and STAT1 Tyr701-phosphorylation induced by IFN-β (which acts through the JAK-STAT pathway to IRF1 induction (53);), thereby confirming the effectiveness of the JAK1/2 inhibitor in blocking STAT1 activation (**Figures 6D–F**). Together our results indicate that the JAK/STAT1 pathway contributes to IRF1 induction by HMPV.

**Figure 7:**
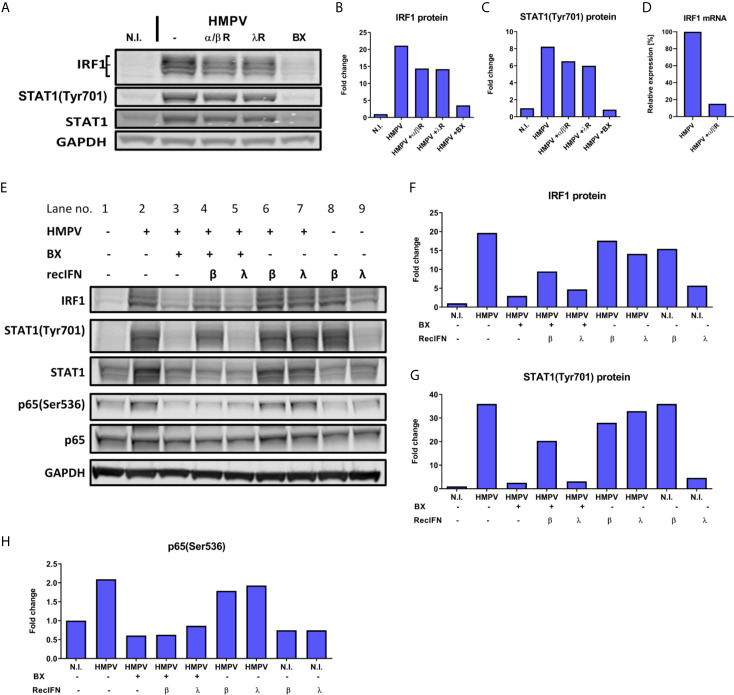
Type I IFN signaling contributes to HMPV-induced expression of IRF1. **(A–D)** Human MDMs were incubated with neutralizing antibodies against IFN-α/βR (α/βR) or IFN-λR (λR), or BX795 (BX) prior to infection with HMPV. Protein levels of IRF1 and p-STAT1(Tyr701) were analyzed by immunoblotting **(A)** and quantified by normalization of band intensities against GAPDH and are presented as fold change relative to uninfected cells (“N.I.”) **(B, C)**. In **(D)** IRF1 mRNA expression was determined in MDMs treated with neutralizing antibodies against IFN-α/βR (α/βR) prior to HMPV infection. IRF1 mRNA levels were normalized to HMPV-infected cells. **(E–H)** Human MDMs were incubated with BX795 prior to infection with HMPV or treatment with medium (N.I.). After 15 h, recombinant IFN-β, IFN-λ1 or medium were added for 3 h. IRF1 **(F)**, p-STAT1(Tyr701) **(G)** and p-p65(Ser536) **(H)** protein levels were analyzed by immunoblotting, quantified by normalization of band intensities against GAPDH and are presented as fold change relative to uninfected cells (“N.I.”). Data are representative of three **(A–D)** or two **(E–H)** independent biological replicates.

IRF1 is strongly induced *via* IFN-β (49, 53). Therefore, we tested if type I and also type III IFNs contribute to IRF1 induction in HMPV-infection by activating IFNAR or IFNLR. Human MDMs were incubated with IFNAR and IFNLR neutralizing antibodies or the TBK1/IKKε inhibitor BX795 (as a control) prior to infection with HMPV. Blocking of either IFNAR or IFNLR partially decreased HMPV-induced IRF1 expression and STAT1 phosphorylation (**Figures 7A–C**). To confirm this result, we confirmed that treatment with anti-IFNAR neutralizing antibodies reduced IRF1 mRNA expression (**Figure 7D**), hence supporting the results obtained for IRF1 protein levels. These results suggest that type I and type III IFN-signaling may contribute to HMPV-stimulated IRF1 expression. To confirm this hypothesis, we tested if exogenous (recombinant) type I or III IFNs could restore HMPV-induced IRF1 expression in MDMs that were treated with BX795 (**Figures 7E, F**). MDMs were treated with BX795 prior to HMPV infection and subsequently with recombinant IFN-β or IFN-λ1. We found that recombinant IFN-β, but not IFN-λ1, was able to partially reverse the inhibitory effect of BX795 on IRF1 expression and STAT1 Tyr701-phosphorylation (**Figure 7E**, lanes 4 and 5). In addition, recombinant IFN-β alone induced IRF1 expression (**Figure 7E**, lanes 1 and 8), while IFN-λ1 barely affected IRF1 (**Figure 7E**, lanes 1 and 9). The increase in IRF1 protein was proportional to levels of phosphorylated STAT1 (**Figures 7F, G**). Moreover, IFN-β (when added alone) induced STAT1 Tyr701-phosphorylation, while IFN-λ1 did not (**Figure 7E**, lanes 8 and 9, **Figure 7G**). These results are consistent with recent results showing that that robust STAT1Tyr701 phosphorylation is required for IRF1 induction and that only IFN-β (not IFN-λ1) enhances IRF1 levels (49). These results indicate that at the tested concentrations and times, IFN-β, but not IFN-λ1, could restore expression of IRF1 in cells lacking TBK1/IKKε kinase activity. Furthermore, when we examined p65 Ser536-phosphorylation (that we have shown contribute to IRF1 induction by HMPV); we found that addition of IFN-β or IFN-λ1 did not significantly change p65 Ser536-phosphorylation (**Figures 7E, H**). Based on these observations we propose that IFN-β-mediated STAT1 activation contributes to IRF1 induction by HMPV.

## Discussion

**Figure 8:**
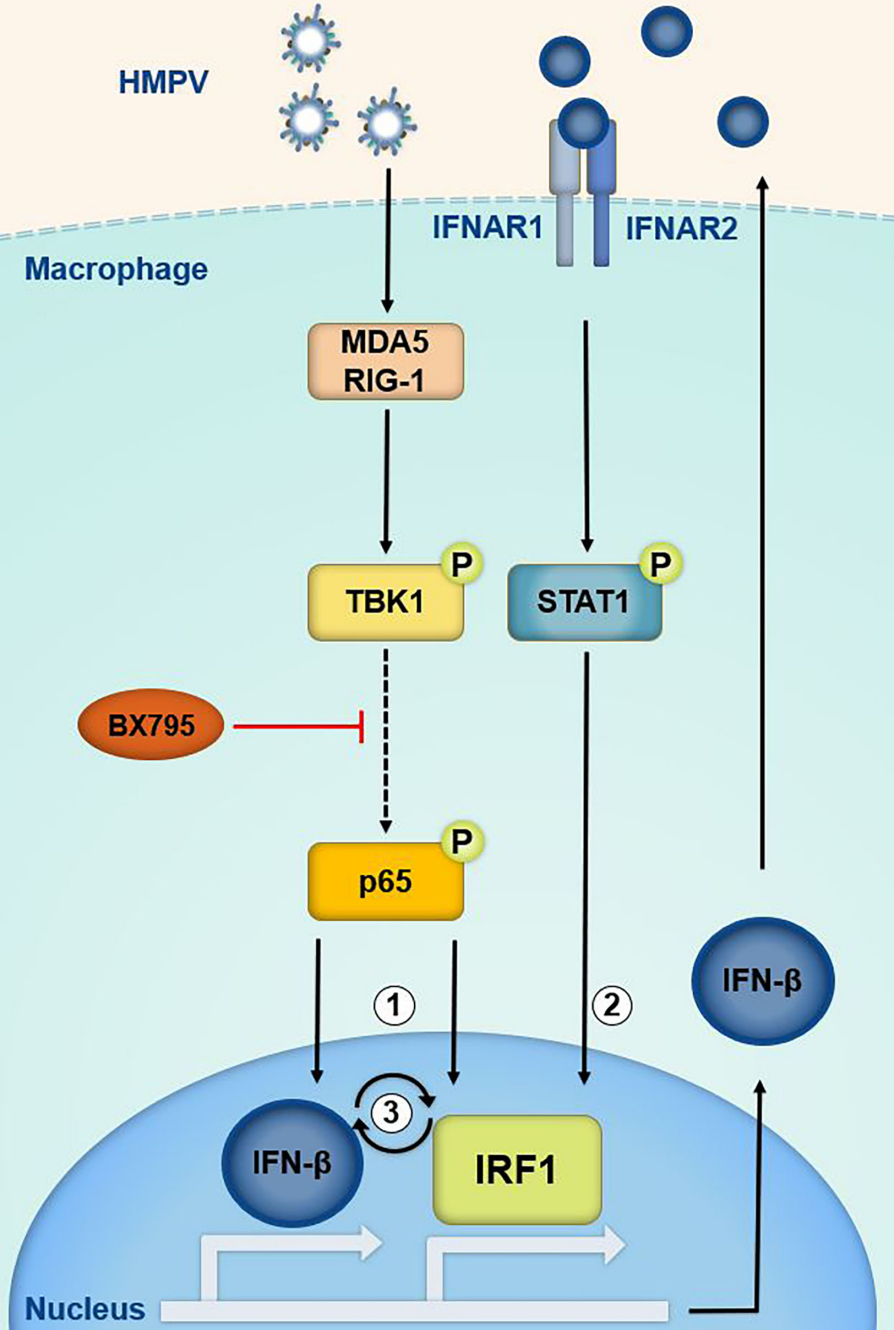
Proposed model of IRF1 transcriptional regulation by TBK1, p65/NF-κB and the IFN-β-STAT1 axis. Based on our results we propose that in HMPV-infected cells TBK1 regulates IRF1 expression *via* the transcription factors p65/NF-κB (1) and STAT1 (2). HMPV may activate TBK1 downstream of RIG-I-like receptors (RLRs) and lead to activation of p65(1) and IFN-β which may stimulate IRF1 expression *via* IFNAR-mediated STAT1 activation (2).BX795 targets both TBK1 and its homolog IKKε. The results suggest that IRF1 is part of an amplifier loop of IFN-β expression (3). Phosphorylation of p65 may contribute to IFN-β induction. IFN-β is believed to be the main driver of IRF1 expression by HMPV in MDMs.

In this study we define a novel IRF1 regulatory link induced by infection with the airway pathogen HMPV. IRF1 controls the expression of multiple genes that are of fundamental importance to host protection against infection and restriction of not only viral, but also bacterial and fungal replication. Examples of IRF1 controlled genes include IFN-λ in viral infections, guanylate-binding proteins (GBPs) in Francisella infection, immunity-related guanosine triphosphatases (IRGs) in Aspergillus fumigatus infection (54, 55) and the immune-responsive gene 1 (IRG1/ACOD1) in infections with Gram-negative bacteria (56, 57). Such an important role of IRF1 in pathogen defense mechanisms underscores the need for detailed knowledge of the mechanisms that govern its expression. Nevertheless, for most pathogens the regulation of IRF1 expression levels throughout the course of microbial infections is not well documented. In this study, we demonstrate that HMPV induces IRF1 *via* a mechanism involving the innate immune kinase TBK1, activation of p65/NF-κB, type I IFN signaling and STAT1 activation leading to efficient IRF1 induction (schematically illustrated in **Figure 8**).

We found that the inhibitor BX795 which in addition to inhibiting TBK1 inhibits its homolog IKKε, blocked HMPV-stimulated p65 Ser536-phosphorylation. In contrast, siTBK1-mediated repression of TBK1 did not reduce phosphorylation of p65, but rather led to increased activation of the NF-κB pathway (determined as increased expression of p65 and the NF-κB regulated genes IκBα, TNF, IL-6). Moreover, levels of IKKε mRNA was increased by HMPV infection and was further increased upon repressing TBK1 by siTBK1-treatment. These results corroborate findings by others showing that for VSV infection, polyIC-treatment and STING activation TBK1 and IKKε act redundantly to elicit NF-κB activation (43, 47). Hence, our results suggest that also for HMPV infection, IKKε may compensate for TBK1 in NF-κB activation, perhaps by phosphorylating Ser536 as has been found in other settings (58). Interestingly, Balka et al. found that for LPS-treatment neither TBK1 nor IKKε was required for p65 phosphorylation, illustrating that the involvement of TBK1 and/or IKKε to p65 phosphorylation is stimulus-specific. Despite that TBK1 appears to be dispensable for HMPV-triggered p65 phosphorylation, siTBK1-treatment partially reduced IRF1 expression stimulated by HMPV (**Figure 3K**). This may reflect that IFN-β, in addition to NF-κB activation, is able to drive HMPV-stimulated IRF1 expression. It will be interesting in future studies to address if TBK1 and IKKε have selective roles in activation of inflammatory/IFN pathways and of different IRF family members in response to HMPV infection.

We found that in contrast to e.g., STAT1 phosphorylation (**Figures 6A, B**), p65 phosphorylation and IκBα induction occurred at the early stages of HMPV infection (**Figures 4A–C**). This induction is TBK1-dependent because it is impaired when TBK1 activity is inhibited (**Figures 4G–I**). Our data may suggest that p65 is involved in IRF1 induction prior to initiation of robust IFN-β-IFNAR-STAT1-mediated signaling. This is based on our observation that little IFN-β were induced at early timepoints while IRF1 mRNA was clearly induced and this induction was inhibited by RelA siRNA-treatment. Hence, we speculate that at the early timepoint, NF-κB may be directly involved in regulation of IRF1, while at later time-points IFN-β exerts the significant contribution to IRF1 induction. Of note, others have identified both an NF-κB binding site and STAT1-binding elements in the IRF1 promoter as being critical for IRF1 induction by TNF and IFN-*γ* (19, 59) and it is possible these promoter elements could be implicated in HMPV-mediated IRF1 induction.

To the best of our knowledge, although it is important in relation to virus infections, the TBK1 (IKKε) -NF-κB-axis has not previously been reported to control IRF1 levels. Nevertheless, it has been reported that NF-κB/RelA is implicated in IRF1 induction following HRV- and RSV infection (21, 22). Hence, HMPV, similarly to the airway viruses HRV and RSV, depends on NF-κB activation for modulating IRF1 levels. As viruses may employ species-specific mechanisms to evade innate immune responses, it is also of interest to determine if TBK1 is also critical for induction of IRF1 in response to HRV and RSV. In HIV infections IRF1 appears to have a dual role: HIV initially upregulates IRF1 in order to initiate HIV replication (60), but at later stages of infection, HIV inhibits IRF1 functions, thereby evading the IRF1-mediated induction of antiviral responses (61). Interestingly, HIV can impair TBK1 activation in human dendritic cells and macrophages (62), but it is currently unknown whether this affects IRF1 expression. Taken together, ours and previously published data strongly suggest that both TBK1 and NF-κB are involved in IRF1 regulation upon infection with a wide set of viral species. Our results implicate NF-κB in the early response to HMPV (i.e. in IRF1 induction). Interestingly, analogous to our results, initial NF-κB activation has been reported to be critical for early induction of antiviral response genes in RSV-infected cells, by triggering an IFN-β-dependent amplification loop (21).

It has been found that TBK1 is required for the regulation of IFN-γ-induced genes by IRF1 and IRF7 (25). Farlik et al. found that TBK1 directly phosphorylated and activated IRF7 in response to IFN-γ. We have earlier shown that HMPV induces IRF7 transcription in MDMs (37). Of interest, it is possible that TBK1 regulates transcription of both IRF1 and IRF7 and that TBK1 (and also IKKε) could regulate IRF7 function not only by activating phosphorylation (as reported by Farlik et al.), but also by stimulating increased IRF7 levels.

Our results suggest that TBK1 (and possibly IKKε) is implicated in p65 Ser536-phosphorylation and NF-κB activation in HMPV-infected cells. Within the scope of this study we did not establish if TBK1 (or IKKε) directly phosphorylates p65 or if this is an indirect process. Several reports indicate that TBK1 may activate NF-κB either directly by phosphorylating p65 at Ser468 and Ser536 (leading to increased transactivation) or indirectly by supporting IKKα/IKKβ activation (by interacting with the upstream TANK-TRAF2-complex) upstream of NF-κB (63–68). Likewise, IKKε have been reported to regulate p65 Ser536-phosphorylation (58). Similarly to our results showing that TBK1 mediates phosphorylation and activation of NF-κB, intracellular sensing of cytosolic dsDNA and herpes simplex virus (HSV; *via* the innate immune adaptor protein STING), leads to NF-κB activation predominantly *via* direct activation of p65 by TBK1 (69).

In summary, our work identifies signaling mechanisms by which IRF1 is regulated by the airway virus HMPV. IRF1 expression and function is important for protective mucosal responses and also for CD8+ T cell responses and our findings on IRF1 regulation may be of relevance for therapeutic purposes or vaccination strategies for airway viruses.

## Data Availability Statement

The raw data supporting the conclusions of this article will be made available by the authors, without undue reservation.

## Author Contributions

SL, VB, AS, and MA conceived the experiments. SL, AS, and KR conducted the experiments and SL, AS, KR, VB, and MA analyzed the results. All authors contributed to the article and approved the submitted version.

## Funding

This work was supported by the Liaison Committee between the Central Norway Regional Health Authority and the Norwegian University of Science and Technology (grant number 46085000), the Faculty of Medicine at the Norwegian University of Science and Technology, the Children’s Clinic, St. Olavs Hospital Trondheim (grant number 16/9564-104/NISLIN), and the MCSA ITN project “INITIATE” (813343).

## Conflict of Interest

The authors declare that the research was conducted in the absence of any commercial or financial relationships that could be construed as a potential conflict of interest.
