# Supplementary material for: Human Metapneumovirus Induces IRF1 via TANK-Binding Kinase 1 and Type I IFN
Source: Front Immunol. 2021 Jun 24;12:563336. doi: 10.3389/fimmu.2021.563336 (PMC8264192; doi:10.3389/fimmu.2021.563336)
Supplement: Supplementary file 1 [file DataSheet_1.docx]

**SUPPLEMENTARY FIGURES**


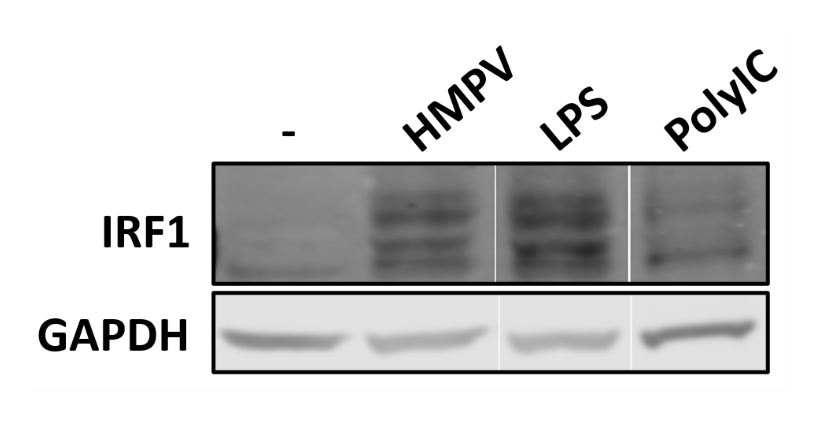


Figure S1: Human MDMs were infected with HMPV (18 hours) or treated with LPS (4 hours, 500 μg/ml) or polyIC (18 hours, 10 μg/ml). Whole cell lysates were prepared and protein levels of IRF1 and GAPDH were analyzed by immunoblotting.


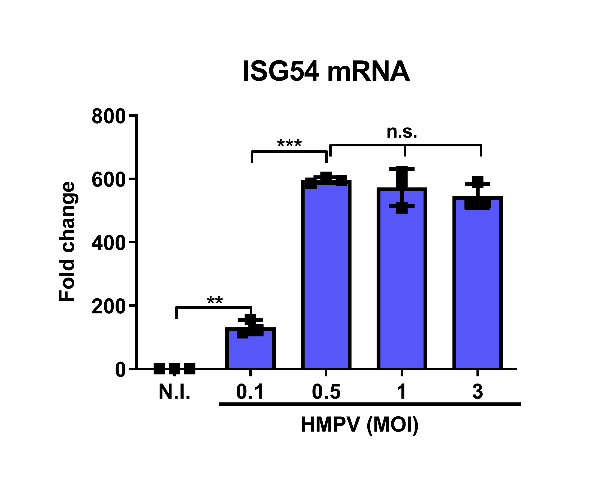


Figure S2: ISG54 expression in MDMs infected with HMPV at different multiplicities of infection for 18 hours. Statistical analysis: One-way ANOVA followed by Tukey’s multiple comparison test: ∗p < 0.05; ∗∗p < 0.01; ∗∗∗p < 0.001.

**
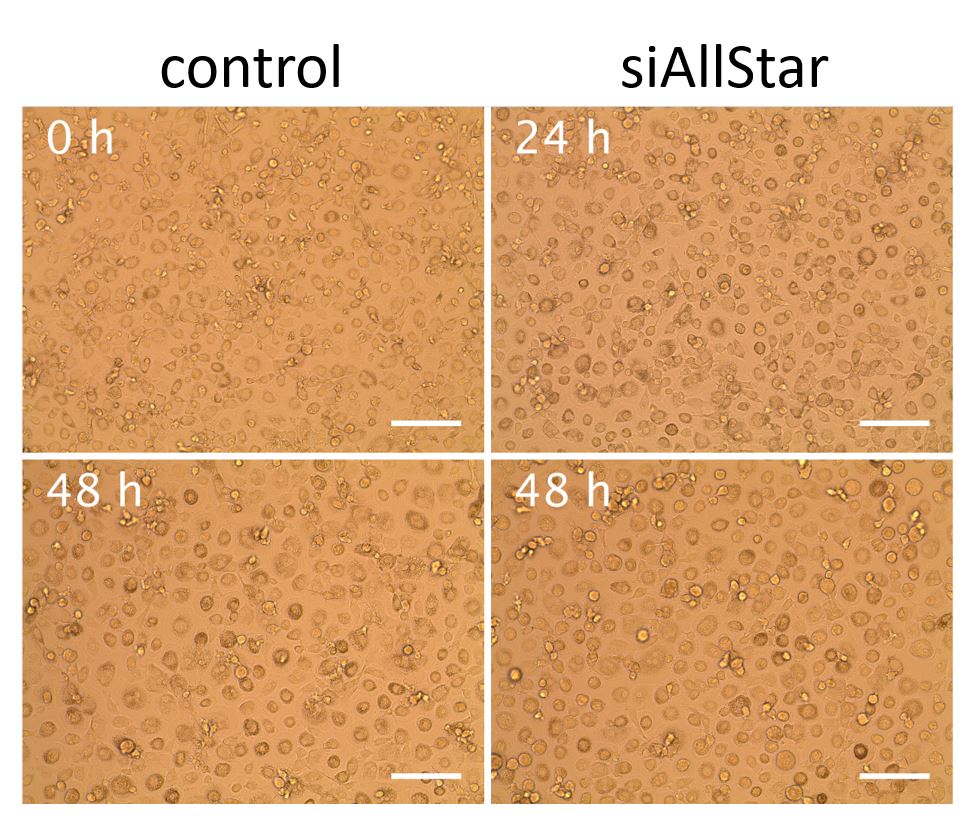
**

Figure S3: Light microscopy images of MDMs showing non-transfected cells (“control”) or cells transfected with 10 nM siAllStar (“siAllStar”) for 24 and 48 hours. The scale bar represents100 µm.


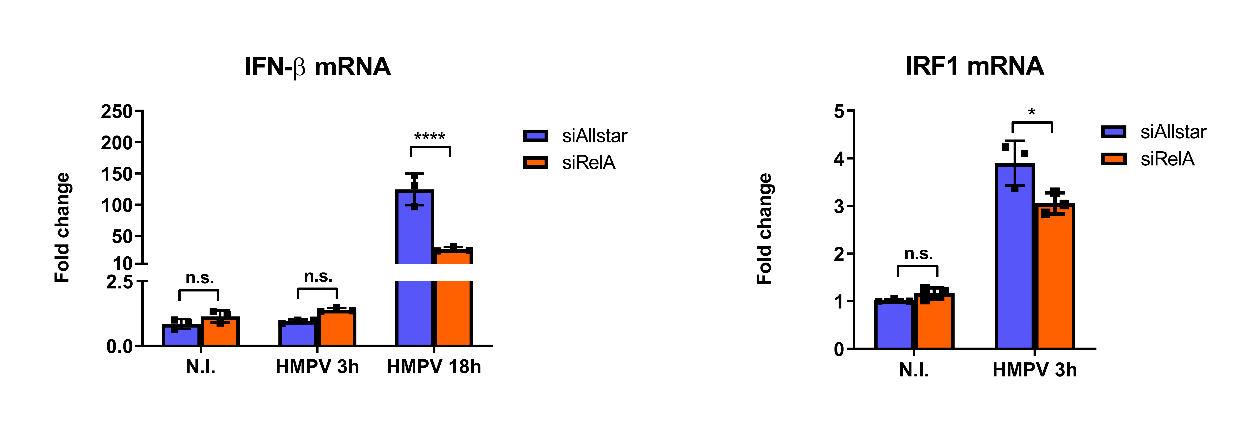


Figure S4: IFN-β and IRF1 mRNA expression in siRelA-transfected MDMs infected with HMPV. Statistical analysis: Two-way ANOVA with Tukey’s honest significance test: ∗p < 0.05; ∗∗p < 0.01; ∗∗∗p < 0.001; ∗∗∗∗p < 0.0001.
